# The roles of small extracellular vesicles as prognostic biomarkers and treatment approaches in triple-negative breast cancer

**DOI:** 10.3389/fonc.2022.998964

**Published:** 2022-09-23

**Authors:** Yueyuan Zhou, Zhongdang Xiao, Wei Zhu

**Affiliations:** ^1^ Department of Clinical Medical Engineering, First Affiliated Hospital of Nanjing Medical University, Nanjing, China; ^2^ State Key Laboratory of Bioelectronics, School of Biological Science and Medical Engineering, Southeast University, Nanjing, China; ^3^ Department of Oncology, First Affiliated Hospital of Nanjing Medical University, Nanjing, China

**Keywords:** small extracellular vesicles, exosomes, triple-negative breast cancer, prognosis, therapeutics, tumor microenvironment

## Abstract

Triple-negative breast cancer (TNBC) is a particularly aggressive and invasive breast cancer subtype and is associated with poor clinical outcomes. Treatment approaches for TNBC remain limited partly due to the lack of expression of well-known molecular targets. Small extracellular vesicles (sEVs) carrying a variety of bioactive contents play an important role in intercellular communications. The biomolecules including nucleic acids, proteins, and metabolites can be transferred locally or systematically to recipient cells and regulate their biological states and are involved in physiological and pathological processes. Recently, despite the extensive attraction to the physiological functions of sEVs, few studies focus on the roles of sEVs in TNBC. In this review, we will summarize the involvement of sEVs in the tumor microenvironment of TNBC. Moreover, we will discuss the potential roles of sEVs as diagnostic markers and treatment therapy in this heterogeneous breast cancer subtype. We finally summarize the clinical application of sEVs in TNBC.

## Introduction

Breast cancer has been globally the most frequent cancer affecting women. Triple-negative breast cancer (TNBC) accounts for approximately 15%–20% of all breast cancer cases and generally demonstrates more aggressive biology with higher grades, more advanced stages at diagnosis, and poorer long-term clinical outcomes compared to other breast cancer subtypes (1–3). It is defined by the absence of expression of the estrogen receptor (ER), progesterone receptor (PR), and human epidermal growth factor 2-receptor (HER2), which are molecular markers to guide treatment and predict prognosis (4–6). Hence, TNBC does not respond to endocrine therapy or other available targeted drugs. Traditional therapeutic approaches such as surgery and systemic chemotherapy are still the first-line treatment for TNBC. However, recurrence and metastases frequently occur in the first 3 years, and the 5-year survival rate is lower than that of other subtypes (7). Therefore, it is urgent to understand the biological profiles of TNBC to develop novel effective therapeutic strategies.

Extracellular vesicles (EVs) can be secreted by nearly all cell types and are found in all biological fluids, including blood, urine, saliva, tears, breast milk, cerebrospinal fluid, amniotic fluid, seminal fluid, and lymphatic fluid (8, 9). They encompass various bioactive molecules such as nucleic acids (mRNA, miRNA, DNA, etc.), lipids, proteins, and even pharmacological compounds (10, 11). Based on particular biogenesis pathways, EVs are classified into three subgroups: endosome-origin exosomes, plasma membrane-derived microvesicles (MVs), and apoptotic bodies (12). Exosomes are secreted and released into the extracellular milieu after the multivesicular body (MVB) fuses with the plasma membrane and released the intraluminal vesicles inside (13–15). MVs are shed from the outward protrusion of the plasma membrane, and apoptotic bodies are released *via* blebbing of the plasma membrane during the late stages of cell death (16–18). Although exosomes are endowed with exquisite activities, they are still lacking experimental support, and there is no consensus on specific markers of EV subpopulations. It was suggested in the MISEV2018 guideline that EVs are defined considering a certain size range as small EVs (<200 nm) and medium/large EVs (>200 nm) (19). Hence, we use the term sEVs to refer to endosome-origin exosomes. Recently, sEVs have emerged as critical mediators of intercellular communication through local and systemic transfer of biological molecules, thereby involved in a variety of physiological and pathological processes. It is suggested that further analysis of sEV contents can unveil the molecular mechanisms involved in tumor progression. Despite limited knowledge of the composition, categories, and functions of sEVs, they still have immense potential as diagnostic biomarkers and therapeutic targets in cancer treatment. In this review, we will briefly report recent studies on sEV communication with the tumor microenvironment in TNBC and summarize the clinical application of sEVs in diagnosis and treatment in TNBC.

## Fields of unsolved problems in triple-negative breast cancer

Although the characterization of TNBC results in the phenotypic absence of ER, PR, and lack of overexpression of HER2, TNBC is a heterogeneous disease comprising various breast cancer subtypes according to the receptor expression profiles. Pathologic and molecular studies revealed that TNBCs correspond to basal-like breast cancers. It has been reported that basal-like markers, including keratin 5, EGFR, and laminin, could be used to classify TNBC (20, 21). However, TNBC is not completely equal to basal-like tumors since 21% of TNBCs are not basal-like, whereas 31% of basal-like are not triple-negative (22). It is necessary to further study the genomic, molecular, and biological bases of TNBC, leading to the identification of novel therapeutic targets. According to gene expression profiles, TNBC was classified into six subtypes, including basal-like 1 (BL1), basal-like 2 (BL2), immunomodulatory (IM), mesenchymal (M), and mesenchymal stem-like (MSL) groups and luminal androgen receptor (LAR) (23). It was demonstrated that the BL1 and BL2 subtypes displayed higher expression of cell cycle and DNA damage response genes, and M and MSL were enriched for epithelial–mesenchymal transition and growth factor signals. The IM subtype was enriched for gene ontologies in immune cell processes, including immune cell signaling (TH1/TH2 pathway, NK cell pathway, B-cell receptor signaling, DC pathway, and T-cell signaling), cytokine signaling (IL-12 and IL-7 pathways), antigen processing, presentation, and key immune signal transduction pathways (such as NF-κB, TNF, and JAK-STAT signaling). The LAR subtype was characterized by androgen receptor (AR) signaling and was associated with decreased relapse-free survival. In addition, it identified four stable TNBC subtypes—LAR, mesenchymal (MES), basal-like immune suppressed (BLIS), and basal-like immune activated (BLIA)—based on mRNA and DNA profiles (24). BLIS tumors have the worst prognoses, while BLIA tumors have the best prognoses. It was revealed that the LAR, MES, BLIS, and BLIA subtypes displayed amplification of specific genes CCND1, EGFR, FGFR2, and CDK1, respectively. These results promote the development of TNBC subtype-specific molecularly targeted therapy and immune treatment.

## Biogenesis and contents of small extracellular vesicles

### Biogenesis and secretion of small extracellular vesicles

sEVs are nano-sized (30–150 nm) vesicles released by almost all cell types and widely present in biological liquids. It was first discovered by the Johnstone team in 1983 that these small particles were associated with the release of transferrin receptors during the maturation of sheep reticulocytes (25) (26). Later, these functional vesicles were defined as exosomes by Johnstone in 1989 (27). sEVs were initially thought to act as the transporter for cells to get rid of metabolic waste (28). It has been recently proved that the secretion of exosomes was an alternative approach to eliminating cellular metabolic products to maintain cellular homeostasis (29, 30). Moreover, growing studies have revealed that sEVs play a critical role in cell-to-cell communication and get involved in both physiological and pathological processes (31–33). Significantly, accumulating evidence demonstrates that tumor-derived sEVs help prepare a suitable microenvironment for cancer cell colonization and distal metastasis (34, 35).

**Figure 1:**
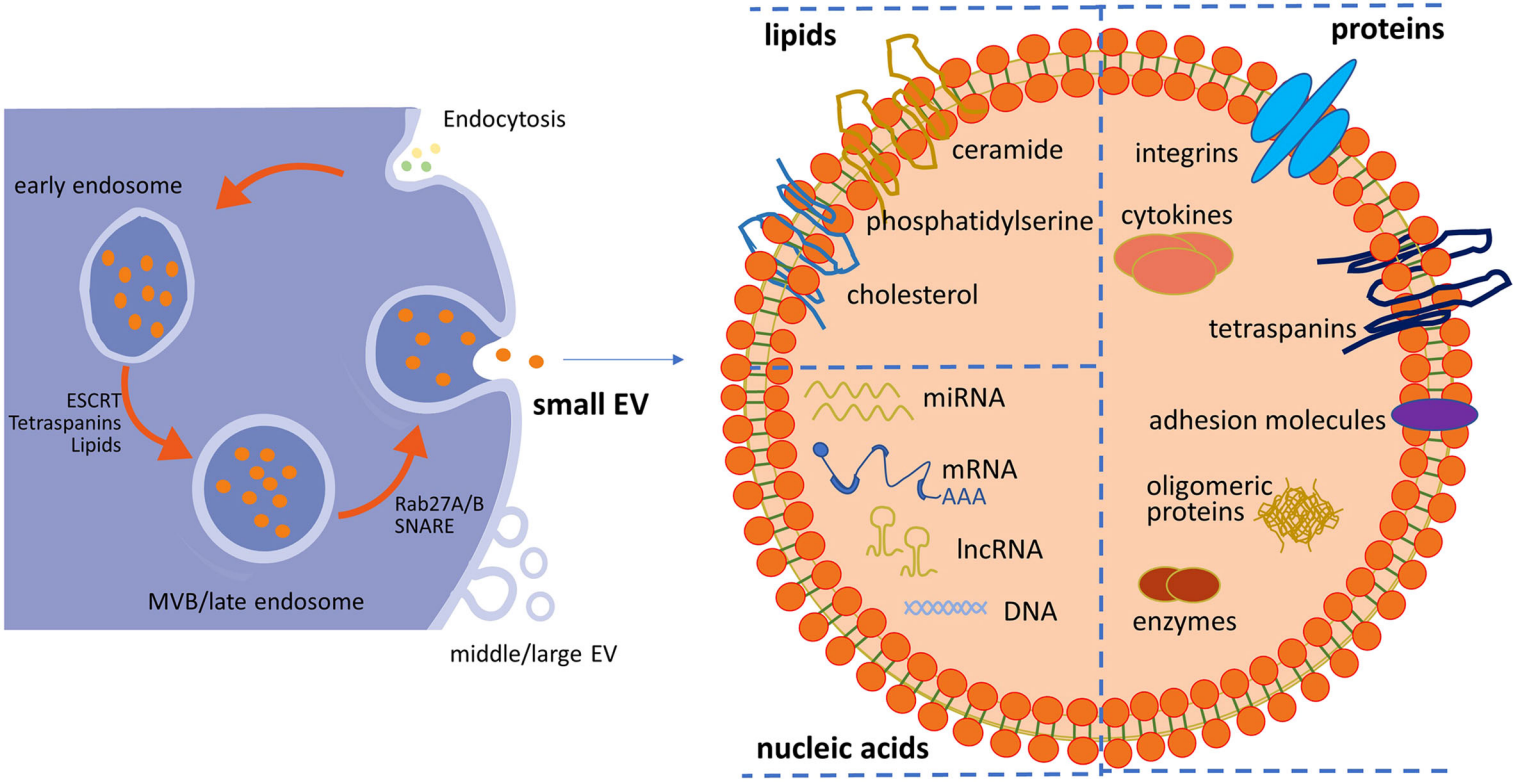
Schematic representation of small EV (sEV) biogenesis and typical structure of sEVs. Within the endosomal system, internalized contents through endocytosis are sorted into early endosomes, which subsequently mature into late endosomes/multivesicular bodies (MVBs). sEVs are released from the fusion of MVBs and the plasma membrane. sEVs accommodate lipids, nucleic acids (DNA, mRNA, and non-coding RNA), and proteins (surface and intra-vesicular molecules). Middle/large EVs bud directly from the plasma membrane. EV, extracellular vesicle.

The release of sEVs requires several cellular steps, including the generation of intraluminal vesicles (ILVs) from MVBs, fusion of MVBs with the plasma membrane, and sorting of distinct sEV cargoes (36–38). As shown in **Figure 1**, sEVs originate from the endosomal pathway by the formation of early endosomes and late endosomes/MVBs. Extracellular fluids and constitutions enter the cells through endocytosis, and the plasma membrane invaginates. Then, internalized contents are sorted into early endosomes. Subsequently, late endosomes/MVBs are formed from early endosomes mediated by endosomal sorting complexes required for transport (ESCRTs) and other associated proteins such as ALIX and CD63 and lipids according to ESCRT-dependent machinery. Finally, MVBs are transported to plasma membrane through the cytoskeletal and microtubule networks and either fuse with lysosomes or autophagosomes to be degraded or fuse with the cell surface, whereby exosomes are secreted (39, 40). Some other studies reported that sEV formation can occur without ESCRTs since multivesicular endosomes containing ILVs existed despite the absence of all four ESCRT complexes (ESCRT-0, ESCRT-I, ESCRT-II, and ESCRT-III) (41–43). The mechanism of sEV biogenesis in an ESCRT-dependent or ESCRT-independent manner may not be completely separated. Furthermore, the cell type and/or cellular homeostasis may have an important influence on the secretion of sEVs.

### Bioactive cargoes of small extracellular vesicles

sEVs accommodate proteins (surface and intra-vesicular molecules), lipids, and nucleic acids (DNA, mRNA, and non-coding RNA), as well as signaling molecules with the lipid bilayer membrane outside (44), as shown in **Figure 1**. It was identified that some common proteins specifically enriched in sEVs, such as CD63, CD9, and CD81, could serve as sEV markers (45). Some other frequent proteins present in sEVs include ESCRT-I-related protein (Tsg101), lysosome-related membrane glycoproteins (LAMP-1 and 2B), MVB-related protein (ALIX-1), heat shock proteins (Hsp60, 70, and 90), adhesion molecules, major histocompatibility molecules (MHC-II), and membrane-binding proteins (annexins) (46–49). These common proteins possess the potential of packaging specific protein molecules into sEVs or carrying targeting molecules on the surface of sEVs, and most of them are transmembrane proteins. It was reported that ALIX recruited ESCRT-III proteins onto late endosomes containing lysobisphosphatidic acid (LBPA) and triggered the formation of ILVs containing CD9, CD81, and CD63 in an ESCRT-independent way (50). Although sEVs contain a common series of components, different results were found in different studies. This may be due to those isolated vesicles being a heterogeneous subpopulation. The heterogeneity is reflective of their cell source, contents, and functional effects on recipient cells. For instance, proteomic analysis of breast cancer cell lines and their sEVs showed that the cell of origin was epithelial-like or mesenchymal-like (51). Proteomic analysis of sEVs isolated from cells with different metastatic propensity demonstrated that the amount and the extent of cancer-related protein cargo vary significantly between non-metastatic and metastatic cell-derived sEVs (52). It was identified that the expression levels of several members of the tetraspanins family (Tetraspanin-14, CD9, CD63, and CD81) were increased in tumor-derived sEVs compared to non-invasive cell line-secreted sEVs. Moreover, sEVs from highly metastatic breast cancer cells induced greater motility (53).

Apart from proteins and peptides, RNA contents, especially miRNAs, have attracted much attention due to their regulatory roles in gene expression. Through a deep sequence of global expression data of a series of cell lines, a subset of miRNAs such as miR-150, miR-142-3p, and miR-451 were generally selected and enriched in sEVs (54). However, some reports have shown that expression levels of sEV-miRNAs differed among various cell lines, as well as the same cell lines under different physiological conditions. The expression level of sEV-miR-21 was lower in the serum of healthy donors than that from glioblastoma patients’ serum (55). Moreover, it was found that miR-451 was highly expressed in sEVs derived from normal cells (e.g., primary T lymphocytes and HMC1 cell) (56, 57). The sorting of miRNAs into exosomes did not randomly occur. It was described that heterogeneous nuclear ribonucleoprotein A2B1 (hnRNPA2B1) was sumoylated and controlled the loading of miRNA into sEVs by binding to them (58). In addition, SYNCRIP, HuR, and major vault protein (MVP) were identified to be involved in the selective incorporation of bioactive cargos into sEVs (59–61). The sEV-lipid composition should be normally consistent with the composition of a lipid bilayer. It was well established that there is an asymmetric distribution of lipid classes in the two leaflets of the plasma membrane, with sphingolipids and phosphatidylcholine (PC) present in the outer leaflet, and other lipid classes located in the inner leaflet (62). The microenvironment and the inherent property may influence the number, contents, and biomarkers of sEVs, but the precise mechanisms of whether and how these bioactive cargoes are sorted and uploaded into sEVs remain unknown.

## Components of small extracellular vesicles involved in triple-negative breast cancer progression

Since sEVs are involved in intercellular communication through transferring content cargoes, they can contribute to tumor microenvironment interactions, including angiogenesis, immune escape, tumor proliferation, invasion, distant metastasis, and drug resistance (63–65). It was reported that the secretion level of sEVs in plasma from patients with breast cancer was higher than that in plasma from healthy controls. The sEV-miRNA expression patterns were different between TNBC and HER2-positive patients, such as miR-335, miR-422a, and miR-628 (66). Furthermore, sEV-miR-374 was associated with higher tumor size in TNBC patients, whereas several miRNAs (miR-185, miR-376a, miR-382, miR-410, miR-433, and miR-628) showed association in HER2-positive patients (66). The excessive release of sEVs can be partly ascribed to the upregulation of TSAP6 transcription by activated p53 in response to DNA damage (67). sEVs derived from more invasive TNBC cell lines significantly increased the proliferation, migration, and invasion capacity of all three recipient cell lines (SKBR3, MDA-MB-231, and HCC1954). These vesicles promoted vasculogenesis and subsequent angiogenesis *via* by stimulating the formation of endothelial tubules (68). sEVs isolated from MDA-MB-231 cells, which are resistant to cisplatin, contained higher expression levels of more than 60 miRNAs compared to those collected from MDA-MB-231 cells. Among these miRNAs, miR-370-3p, miR-423-5p, and miR-373 were the most differentially expressed miRNAs (69). These functional miRNAs may have differential expression levels and possess the potential as diagnostic tools and therapeutic interventions.

In addition to the delivery of miRNAs in sEVs, some sEV proteins were found to participate in cancer progression and metastasis. It was revealed that Rab27A promoted the invasive and pulmonary metastatic potentials of TNBC MDA-MB-231 and HER2+ MDA-MB-435 breast cancer cells (70). Consistently, Rab27a was found to promote tumor progression in part by inducing the secretion of sEVs (71). Treatment with sEVs derived from MDA-MB-231 cells could also promote breast cancer cells migrating to the zebrafish tail, which was mediated by overexpression of thrombospondin-1 (TSP1) suppressing intercellular junction molecules (72). For bone metastasis, sEV release of L-plastin and peroxiredoxin-4 (PRDX4) from MDA-MB-231 cells mediated breast cancer-induced osteolysis. The specific mechanism was that L-plastin stimulated osteoclast formation from late osteoclast precursors in the absence of RANKL through stimulation of calcium oscillations and nuclear translocation of NFATc1 transcription factor (73). It was also proved that CD151 transferred by sEVs derived from MDA-MB-231 helped enhance TNBC cell line (MDA-MB-231 and MDA-MB-468) migration and invasion abilities, and sEV-CD151 was significantly enriched in the serum from TNBC patients (74). These results offer evidence that exosomes have a pathophysiological role in TNBC.

Other components of sEVs were reported to participate in the tumor microenvironment as well. For instance, long non-coding RNAs (LncRNAs) are non-coding RNAs with more than 200 nucleotides that lack protein-coding capability due to the absence of open reading frames and start and stop codons (75). Enhanced expression levels of LncRNA metastasis-associated lung adenocarcinoma transcript 1 (MALAT1) were found in breast cancer cells and secreted sEVs. sEV-MALAT1 from cancer cells could significantly induce TNBC cell proliferation (76). Circular RNAs (CircRNAs) are formed by exon back-splicing by connecting the downstream 5′ splicing site to the upstream 3′ splicing site, and they are characterized by evolutional conservation, high stability, and insensitivity to exoribonucleases (77, 78). It was reported that circular RNA arose from HIF1A gene that was overexpressed in breast cancer tissues and sEVs from the plasma of breast cancer patients. CircHIF1A was demonstrated to enhance TNBC cell growth and migration through modulation of miR-149-5p and NFIB and further promote TNBC progression and metastasis (79). Although a variety of progress has been made in the study of sEVs in recent years, its function in TNBC tumorigenesis is still beginning to be understood. Further, the specific role of sEVs in the TNBC microenvironment should be identified, thereby better applying sEVs in clinical treatment.

## The potential for clinical application of small extracellular vesicles in triple-negative breast cancer

### Isolation and characterization of small extracellular vesicles

sEVs contain various cargoes (DNA, RNA, protein, lipid, and metabolites) and are enriched with specific cancer-associated contents. They are detected to be relatively stable in biological fluids, such as plasma, urine, semen, saliva, amniotic fluid, and tears. The concentration of sEVs was reported to be higher in the systemic circulation of patients with ovarian, breast, and pancreatic cancers (80, 81). sEVs inherit distinct molecules from their cell source and mimic the behavior of the parental cells. Therefore, sEVs have attracted tremendous interest in the biomarker research field.

To be utilized as diagnostic biomarkers, the first key point is standard isolation and characterization of sEVs. A variety of methods have been proposed to isolate and purify sEVs, and they are generally developed based on isolation by size, immunoaffinity capture, and precipitation (**Table 1**). However, these methods fail to exclusively isolate sEVs and typically result in complex mixtures of sEVs and other components of extracellular space. Among these methods, differential ultracentrifugation was the first method to be used for sEV isolation and remains the gold standard for sEV isolation (82) (83). The representative protocol for sEV isolation is differential ultracentrifugation. The yield can be increased *via* ultracentrifugation at the spin of 100,000 × *g* for a longer time, but ultracentrifugation for a too long time (>4 h) may induce mechanical damage to sEVs and contamination of soluble proteins in the final pellets (84). Differential ultracentrifugation does not require too much technical expertise and sample pretreatment, although it costs time and a large volume of samples or cell culture medium. In order to collect sEVs from a relatively small volume of clinical samples such as plasma, size exclusion chromatography (SEC) is a more clinical setting-friendly option since it allows for sEV isolation from 150 μl to 10 ml of biofluid with resins of selected size (85) (86). Moreover, SEC can protect sEVs from aggregation and improve the removal of protein contaminants (87). In addition, size exclusion chromatography is applied as the purification step after ultracentrifugation methods. An optimized isolation and purification protocol for collected high yields of sEVs from blood was determined as below: firstly, the plasma or serum was centrifuged at 18,000 × *g* for 30 min at 4°C. Then, proteinase K was added to the supernatant (25 μg per 10 mg total proteins of sEV sample) to decrease the amount of albumin and apolipoproteins A-1 and B. Finally, a SEC resin with a molecular weight cutoff (MWCO) of 700 kDa was used to further clear small peptides or proteins (88). Microfluidic isolation can isolate sEVs based on their physical and biochemical properties at the same time. It requires a smaller volume of samples and can be developed into innovative separation, which makes clinical use of sEVs more feasible (89). Immuno-based microfluidic isolation is dependent on the interaction between a membrane-binding protein on sEVs and an antibody against the protein, which is immobilized on a microfluidic chip. The predominant advantage of this method is that it requires the smallest volume of the plasma/serum, the least amount of time, minimal expertise, and the least cost to date.

**Table 1:** Comparison of separation technologies of sEVs.

Isolation method	Advantages	Limitation		
Ultracentrifugation	Large sample volume, high yields		Long operation time, equipment requirement, mechanical damage, and protein contamination
Filtration	Fast process, low equipment requirement		sEV damage due to shear stress and loss due to membrane trapping
Size exclusion chromatography	High purity, fast preparation, good reproducibility		Combination with sEV enrichment
Microfluidics	High efficiency, low cost, high sample capacity		Low specificity, contamination of protein and polymeric materials
Immunoaffinity capture	High specificity, high purity		High cost, low sample capacity, and low yields

sEVs, small extracellular vesicles.

The identification and characterization of sEVs are divided into two types: physical analysis and chemical or compositional analysis. Physical analysis determines particle size and concentration through nanoparticle tracking analysis (NTA), transmission electron microscopy (TEM), scanning electron microscopy (SEM), atomic force microscopy (AFM), and dynamic light scattering (DLS). Chemical or compositional analysis evaluates specific contents such as miRNA and protein *via* sequencing, immunoblotting, and staining. The obstacle is still how to differentiate subpopulations of extracellular vesicles with distinct markers and sizes. What makes it more challenging is the fact that the isolation method affects the profiles of sEVs.

### Small extracellular vesicles as diagnostic and prognostic biomarkers for triple-negative breast cancer

Following further knowledge of the molecular heterogeneity of TNBC, liquid biopsy has attracted much attention since traditional cancer detection approaches showed weakness in the analysis of the genomic landscape of TNBC. Additionally, liquid biopsy can monitor cancer progress or clinical outcome after treatment in a non-invasive manner. A series of components are released in the tumor microenvironment, for instance, circulating tumor cells (CTCs), cell-free DNA, and EVs in blood circulation (90). sEVs display superiority over other components, as they generally exist in biological liquids and can be easily isolated, stored, and transported. Furthermore, abundant sEV inclusions allow for diverse expression profile analysis.

sEVs collected from breast cancer patients have distinct protein and RNA contents as compared to sEVs derived from healthy donors. As listed in **Table 2**, it was reported that the serum level of sEV-miR-373 was significantly upregulated in patients with TNBC compared to other breast cancer subtypes, and sEV-miR-372 was increased in breast cancer patients than that in healthy controls (91). Subsequently, functional analyses revealed that miR-373 might downregulate the protein expression level of ER and inhibit apoptosis *via* camptothecin. Interestingly, it was found that the majority of miRNAs detectable in plasma were concentrated in sEVs. The high miRNA concentration observed in sEVs may be due to sEV protection from digestion by RNase. The number of sEVs from plasma was obviously larger in TNBC and HER2+ patients than that in healthy donors (66). A panel of sEV-miR-335, miR-628, and miR-422a could discriminate between TNBC and HER2+ patients. Moreover, in TNBC patients, sEV-miR-374 showed an association with tumor size. These findings suggest a combination of sEV-serum miRNA levels as TNBC-specific markers.

**Table 2:** sEV contents as biomarkers for BC and TNBC.

Source	Species	Cargo	Reported effects	References
TNBC serum	RNA	miR-373, miR272 ↑	Decrease ER, inhibit apoptosis	(91)
TNBC plasma		miR-335, miR-628, miR-422a ↑	Promote proliferation	(66)
TNBC serum		lnc-SUMO1P3 ↑	Correlate with lymph vascular invasion, lymph node metastasis	(92)
TNBC serum		lnc-XIST ↑	Correlate with TNBC recurrence	(93)
BC plasma	Protein	Phosphoproteins ↑	Participate in phosphorylation	(94)
Biological fluids		Lipid raft proteins	Function in membrane signaling and trafficking	(95, 96)

sEVs, small extracellular vesicles; BC, breast cancer; TNBC, triple-negative breast cancer; ER, estrogen receptor.

↑ means increase.

sEV-LncRNA has been revealed to be associated with tumor development and cancer progression. The well-studied LncRNA, HOX transcript antisense intergenic RNA (HOTAIR), was detected in sEVs derived from breast cancer patients, and the expression level of HOTAIR was positively correlated with HER2 in tumor tissues (97). These results suggested HOTAIR as a novel liquid biopsy biomarker for breast cancer. The expression level of serum sEV-LncRNA small ubiquitin-like pseudogene 3 (SUMO1P3) was significantly higher in patients with TNBC compared to that in patients with non-TNBC, patients with benign breast disease, and healthy controls (92). Serum sEV-SUMO1P3 was closely correlated with lymph vascular invasion, lymph node metastasis, and histological grade and positively corresponded to overall survival. Furthermore, serum sEV-SUMO1P3 levels were markedly decreased in chemosensitive cases. These findings showed the potential of serum sEV-LncRNA SUMO1P3 as an independent prognostic factor for TNBC. It was identified that serum sEV-LncRNA XIST obviously increased in TNBC recurrence and could distinguish TNBC patients from healthy controls through receiver operating characteristic (ROC) curve analysis, implying the function of sEV-XIST as a diagnostic and prognostic biomarker for TNBC (93). However, the underlying molecular mechanisms remain largely unknown, and further supporting evidence is required from larger independent studies.

In addition to RNAs, sEV proteins possess unique features as biomarkers. Specifically, phosphoproteins have the potential as cancer markers because protein phosphorylation is involved in almost all cellular processes (98, 99). It was identified that the expression levels of 144 phosphoproteins were significantly higher in plasma sEVs from patients diagnosed with breast cancer than those in healthy controls through label-free quantitative phosphoproteomics (94). Moreover, lipid rafts proteins are also enriched in the sEV membrane since they organize and stabilize the liquid-ordered regions of the membrane and compartmentalize the processes of intracellular signaling, creating the signaling platforms where interacting components (receptors, effector proteins, and coupling factors) are colocalized in spatial proximity (95, 96). A high abundance of stomatin was shown in sEVs derived from biological fluids, including blood plasma, ascitic fluids, and uterine flushings (100). The expression level of stomatin protein in sEVs from different sources corresponds well to that of CD9, whereas the level of caveolin-1 varies drastically depending on cell type.

The first commercial sEV-based ExoDx™ Prostate (IntelliScore) (EPI) test has been applied for prostate cancer in 2016 (101, 102). This novel non-invasive urine test assessed the expression level of three sEV-RNA transcripts (ERG, PCA3, and SPDEF) for the risk management of men over 50 years of age with PSA level in the “gray zone” of 2–10 ng/ml. The test was validated at a cut point of 15.6 to rule out high-grade prostate cancer and would avoid 27% of invasive biopsies. This sEV-based test has been included in the National Comprehensive Cancer Network guidelines for early prostate cancer detection. We believe this milestone product will promote the development of sEV-based early cancer diagnosis.

### Small extracellular vesicles as drug delivery system for treatment approach

sEVs are enriched in biological fluids (such as blood, saliva, and urine), encapsulated with various bioactive cargoes, and mediate intercellular communication by delivering cargoes from parental cells to recipient cells. There is compelling evidence that sEVs can penetrate through the hematoencephalic barrier, keep stability in long circulation, and maintain specific targeting effects (103–105). sEVs derived from different sources carry diverse surface molecules and contents and exert various effects. sEVs serving as drug delivery vehicles should have specific quality standards including size, yield, surface protein, and intracavitary composition.

Mesenchymal stem cells (MSCs) have advantages in terms of ease of expansion, harvesting, and low immunogenic ability. As shown in **Table 3**, sEVs from MSCs derived from human induced pluripotent stem cells (iPSCs) were loaded with the chemotherapeutic drug doxorubicin (DOX) and showed superior cytotoxic effects on doxorubicin-resistant TNBC cells compared with free or liposomal DOX (106). These vesicles significantly inhibited metastases in TNBC mouse models without detectable immunogenicity. sEVs inherit the essential immunostimulatory faculties from parental dendritic cells (DCs) and lack the risk of *in vivo* replication. It was initially reported that sEVs derived from DCs modified with RVG-targeted Lamp2b peptide delivered siRNA to neurons, microglia, and oligodendrocytes in the mouse brain and strongly downregulated the expression of BACE1 mRNA and protein (107). These results suggested the therapeutic benefit of DC exosomes in Alzheimer’s disease since BACE1 is responsible for the N-terminal cleavage of amyloid precursor protein that produces the aggregate-forming β-amyloid peptide in Alzheimer’s disease pathogenesis (112). Macrophages are a group of heterogeneous cells that can be phenotypically polarized in the tumor microenvironment to initiate the adaptive immune response (113, 114). Feng et al. modified macrophage-derived sEV-coated nanoparticles carrying DOX for targeted chemotherapy of TNBC (108). It was firstly reported that sEVs from macrophages could penetrate the blood–brain barrier without targeting modification (115). The expression of the integrin lymphocyte function-associated antigen 1 (LFA-1) and intercellular adhesion molecule-1 (ICAM-1) in naïve macrophage sEVs mediated the uptake of exosomes in brain endothelial cells, thereby helping sEVs deliver brain-derived neurotrophic factor (BNDF) to the brain, especially in the presence of brain inflammation. Since patients with TNBC are at a high risk of incidence of brain metastases, the natural crossing blood–brain barrier feature of sEVs holds the promise of improving the survival and life quality of TNBC patients with brain metastasis (110, 116). Compared with sEVs derived from non-cancerous cells, sEVs that originated from tumor cells specifically carry tumor antigens and costimulatory molecules and can lead to an anti-tumor immune response (109). It was reported that sEV-like nanovesicles developed from metastatic breast cancer 4T1 cells could effectively deliver doxorubicin to the lung of the mouse model and inhibited breast cancer lung metastasis (111).

**Table 3:** sEVs derived from different types of origins served as drug delivery system.

Source		Cargo			Disease	Reference
Cell source	MSCs		Doxorubicin	TNBC		(106)
	DCs	siRNA		Alzheimer’s disease		(107)
	Macrophages	Doxorubicin		TNBC		(108)
	Tumor cells	Doxorubicin		Breast cancer		(109)
Acellular origin	Saliva	mRNA		Wound healing		(110)
	Plasma	Quercetin		Alzheimer’s disease		(109)
	Milk	Withaferin A, paclitaxel, docetaxel		Lung cancer		(111)

sEVs, small extracellular vesicles; MSCs, mesenchymal stem cells; DCs, dendritic cells; TNBC, triple-negative breast cancer.

Apart from cell-derived sEVs, these vesicles from biological liquid also possessed advantages as a drug delivery system. For instance, saliva sEVs accelerated wound healing by transferring UBE20, which enhanced the proliferation, migration, and angiogenesis of human umbilical vein endothelial cells (HUVECs) (117). Meanwhile, it was found that saliva sEVs have unique features including distinct elastic properties and substructures carrying specific transmembrane receptors (118). It was firstly proved by Valadi’s group that plasma sEVs were uploaded siRNA through chemical transfection and electroporation and delivered the siRNA to monocytes and lymphocytes, leading to gene silencing of mitogen-activated protein kinase 1 (11). Plasma sEVs were lately packed with quercetin, inhibited the activity of CDK5 and decreased tau protein hyperphosphorylation, and attenuated neurodegeneration by reducing the apoptosis of neuron cells and improving memory and spatial learning (119). These findings suggest that sEVs isolated from plasma can be applied as a delivery vehicle of exogenous nucleic acids and chemical drugs for better treatment of central neurological diseases *via* crossing the blood–brain barrier. Bovine milk is generally considered to be a potentially scalable source of sEVs serving as drug delivery vehicles. It was investigated that milk sEVs could encapsulate with both hydrophilic and lipophilic small molecule drugs and exhibit tumor targetability without adverse immune and inflammatory responses (120). In addition to bovine milk exosomes, human breast milk-derived sEVs (HBM-sEVs) also have the potential to be utilized for drug delivery. HBM-sEVs were reported to protect the intestine from damage through intervening intestinal immune response (121) (122). It is worth noting that HBM-sEVs promoted cell proliferation of normal colon epithelial cells, whereas they exerted no beneficial effects on tumor cells (123). These results revealed that HBM-sEVs possess superiorities over other types of sEVs due to their intestinal protection and transferring anti-tumor drug without inducing tumor cell proliferation.

**Figure 2:**
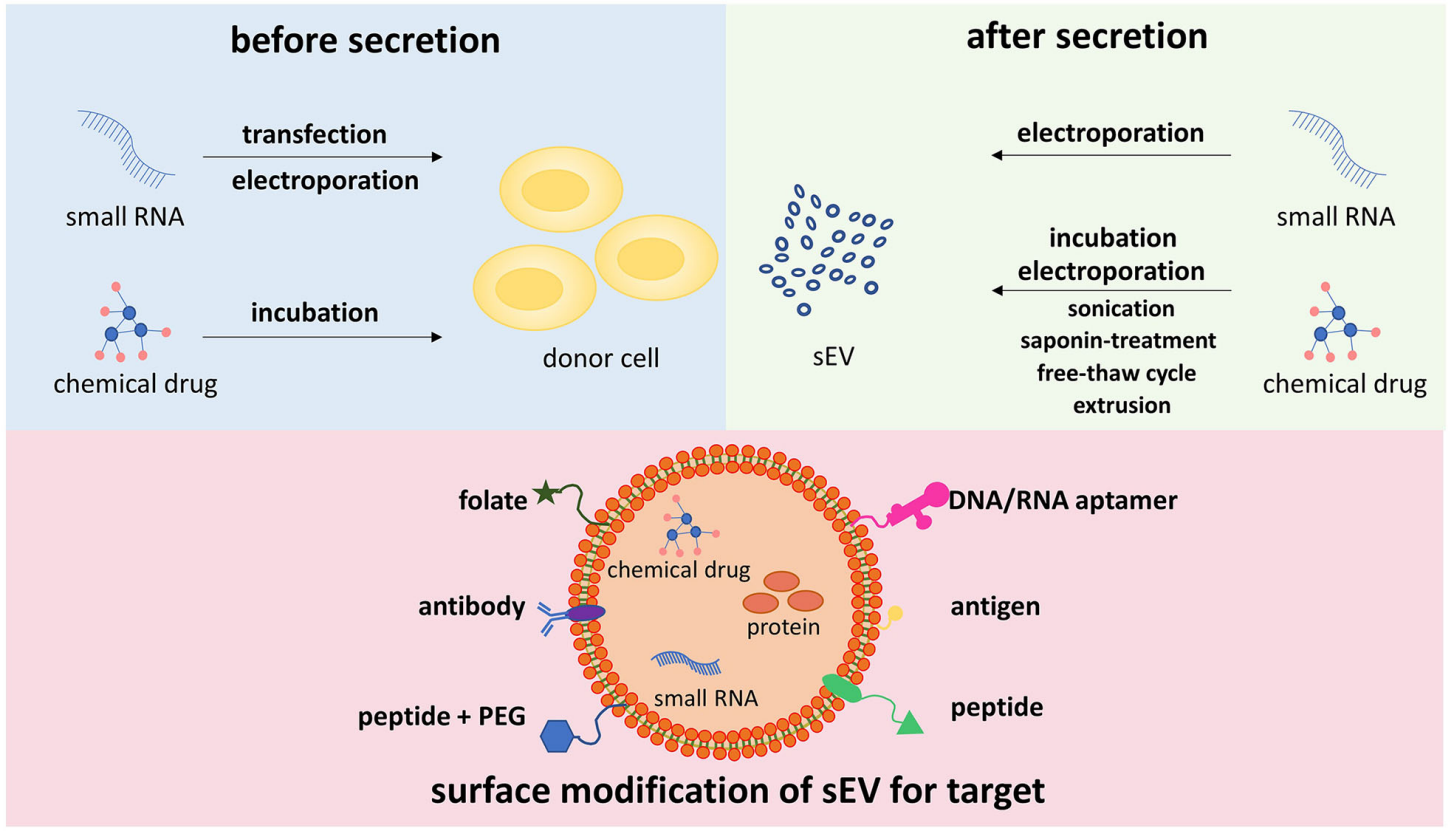
Different methods for drug loading into sEVs, and surface engineering for targeting specificity sEVs can be uploaded with drugs *via* two types of methods, introducing drugs into cell origin before sEV secretion and loading drugs into sEVs directly. The former approach includes transfection, electroporation, and incubation. The latter approach consists of electroporation, incubation, sonication, saponin treatment, free–thaw cycle, extrusion, and so on. To enhance targeting activity, the surface of sEVs is modified to express affinity molecules, such as peptides, DNA/RNA aptamers, folate, antibodies, and antigens. sEVs, small extracellular vesicles.

sEVs can be uploaded with drugs (chemical molecules and/or RNAs) through different techniques, which are mainly discussed in two manners (**Figure 2**). One approach is to load drugs into the donor cells of sEVs, and then the drugs are sorted into sEVs. There are two representative methods, including transfection and electroporation for RNAs and co-incubation for chemical drugs (107–125). Transfection ensures that target miRNA or siRNA is encapsulated into sEVs and released after sEV internalization by recipient cells. It was previously reported that donor cells, HEK293, and COS-7 cells were transfected with miRNA, which targeted EGFR, and secreted sEVs overexpressing the miRNA (126). However, the disadvantage of transfection is the unstable encapsulation efficiency of RNA, which may have an influence on downstream targeting effects. The basic principle of electroporation is that the application of short, high-voltage pulses penetrates the lipid membrane of cells or sEVs, and then the drugs are loaded into sEVs inside (127). Co-incubation is another method to load drugs especially small chemical molecules into sEVs. By exposure of MSCs to high concentrations of paclitaxel (PTX), PTX was incorporated into MSCs and subsequently released sEVs (125). However, after incubation of parental cells with drugs, the synthesis and secretion of drug-carrying sEVs are difficult to be managed. Another way is to introduce drugs directly into sEVs after they are released and isolated, consisting of co-incubation, electroporation, sonication, saponin treatment, extrusion, and freeze–thaw cycles. Compared to the incubation mentioned above, sEVs can be mixed with drugs directly, which is simpler and more effective. Based on the lipophilicity of PTX and passive diffusion, PTX was loaded into sEVs directly by co-incubation with relatively high loading efficiency (128). It was demonstrated that PAK4-specific siRNA was encapsulated into sEVs derived from PANC-1 cells through electroporation, and the encapsulation efficiency and the loading efficiency were 10%–20% and 5%, respectively (129). It is inferred that the aggregation of sEVs during electroporation and the intraluminal space within sEVs, which is occupied by siRNA, is fully saturated, leading to the lower encapsulation efficiency of electroporation (130, 131). In addition to co-incubation and electroporation, there are several other approaches for drug loading in sEVs after their releases, such as sonication, saponin treatment, free–thaw cycle, and extrusion (132). Despite the natural origin of sEVs endowed with homing features, sEVs can be surface-engineered to enhance targeting specificity. As shown in **Figure 2**, genetic modification links antibodies, peptides, DNA/RNA aptamers, and tumor antigens with the transmembrane domain. Tian et al. engineered immature DC-derived sEVs with αv integrin-specific iRGD peptides and uploaded DOX into these vesicles through electroporation (133). These modified sEVs showed highly efficient targeting and DOX delivery to αv integrin-positive breast cancer cells, leading to the inhibition of tumor growth without overt toxicity. In another study, sEVs were labeled with folate to target TNBC cells with overexpression of folate receptors, and these sEVs exerted a better inhibitory effect on the proliferation and migration of TNBC cells (134). Targeting sEV-based drug delivery system helps generate sEVs with a high yield and low toxicity.

A variety of administration approaches have been exploited to deliver sEVs to target tissues in different disease models, such as direct injection, intravenous injection, intraperitoneal injection, oral administration, and, recently, inhalation. Direct injection showed high efficiency in inhibiting the proliferation of cancer cells and decreasing tumor mass (135). However, direct injection was more invasive than a systemic approach (intravenous injection) (136). Intravenous injection is generally selected for sEV delivery; however, the clearance of this route is rapid (137). It was fluorescently detected that exosomes were predominantly accumulated in liver, lung, kidney, and splenic tissues after intravenous injection (138). With the use of chemiluminescence, sEVs were detected primarily in the liver and the lung, and the signal was retained in the lung longer than that in other organs (137). Moreover, sEVs were found to distribute to the brain and intestines after intranasal administration (138). When sEVs were modified with neuron-specific targeting peptides, they were detected in the central nervous system after intravenous injection (107). Inhaled sEV treatment provides beneficial effects for inflammatory lung diseases including asthma, chronic obstructive pulmonary disease (COPD), acute respiratory distress syndrome (ARDS), and COVID-19 since it can prevent first-pass hepatic metabolism, improve drug solubility and distribution, and reduce drug side effects (139). The outstanding advantages of sEVs as drug delivery systems lie in their biological origin, which is strongly associated with good biocompatibility. However, some critical questions remain to be answered before clinical application. One of the major challenges is the large-scale standardized production of therapeutic sEVs, which include the origin choice, isolation and purification method, external modification and drug encapsulation, storage, and transportation.

### New advances in small extracellular vesicle-based therapy for triple-negative breast cancer

sEVs as nano-sized drug delivery vehicles have attracted attention in TNBC. Some TNBC cells have been demonstrated to be sensitive to erastin-induced reactive oxygen species (ROS)-dependent ferroptosis, followed by significant suppression of cell proliferation and migration (140, 141). However, the poor water solubility of erastin results in low absorption, and renal toxicity limited its clinical application (142). It was reported that erastin was loaded into folate-modified sEVs and can be successfully transported to TNBC tumor sites, thereby increasing the inhibition rate of erastin on the cytotoxicity, proliferation, and migration of TNBC MDA-MB-231 cells (134). Erastin carried by folate-vectorized sEVs caused more ferroptosis with intracellular depletion of glutathione and reactive oxygen species overgeneration than erastin carried by natural sEVs and free erastin (134). The results revealed that erastin loaded in a sEV-targeted delivery system increased the uptake efficiency of erastin into TNBC cells with a longer duration of action and higher activity. Genetically engineered chimeric antigen receptor T cell (CAR-T) therapy has rapidly developed into a powerful and innovative treatment for cancer patients (143, 144). Despite the unprecedented success of CAR-T therapy in B-cell leukemia or lymphoma, many challenges limited its therapeutic effects in solid tumors such as dose-dependent systemic toxicity. sEVs derived from mesothelin-targeted CAR-T cells inherited surface expression of the CARs and CD3 from parental CAR-T cells and strongly inhibited the growth of both endogenous and exogenous mesothelin-positive TNBC cells (145). The cytotoxicity against TNBC cells of CAR-T-derived-sEVs is exerted through the release of effector molecules perforin and granzyme B with low toxicity *in vivo*. Hence, it was suggested that CAR-T-derived sEVs as a cell-free alternative therapy with efficient cytotoxicity and favorable safety. Immune checkpoints play critical roles in tumor immune surveillance. After analysis of the expression pattern of immune checkpoints using ICP array, sEVs derived from activated tumor-associated effector T cells carry membrane-bound PD-1 (146). Furthermore, they enhanced the cytotoxicity of T cells against TNBC cells by occupying PD-L1 and attenuating subsequent T-cell dysfunction. Altogether, activated T cells in TNBC tumor microenvironment inhibited tumor growth and enhanced anti-tumor immunity. Not only T cell-derived sEVs but also other types of immune cell-derived sEVs can target tumor cells. A macrophage-secreted sEV-based nanosystem was developed, which was modified with peptide targeting the mesenchymal–epithelial transition factor (overexpressed by TNBC cells) and loaded DOX (108). These engineered sEVs obviously prolonged the circulation time of DOX, specifically targeted tumors, and promoted apoptosis of tumor cells with low hepatotoxicity.

In addition to target-modified sEVs, sEVs secreted from breast cancer cells were demonstrated to exhibit excellent lung targeting properties owing to their functional surface integrins, which co-located in the laminin-rich lung microenvironment (147). In order to utilize the natural targeting characteristic, the membrane of sEVs derived from breast cancer cells was extracted and wrapped around cationic bovine serum albumin-conjugated S100A4 siRNA (148). These biomimetic nanoparticles displayed gene-silencing effects on S100A4, which was an important metastasis-related protein that promotes tumor progression and metastasis and suppressed postoperative breast cancer metastasis (149, 150). TNBC cell-derived sEVs were reported to be utilized as a DC-primed vaccine to induce antitumor immunity (151). In specific, sEVs originating from MDA-MB-231 cells were genetically engineered to overexpress α-lactalbumin, which was expressed in the majority of human breast cancers, hence showing enhanced tumor-targeting capability and immunogenicity. The sEVs were subsequently loaded with the immunogenic cell death (ICD) inducers human neutrophil elastase (ELANE) and Hiltonol. This combined delivery system activated DCs *in situ* and cross-primed tumor-reactive CD8^+^ T-cell responses, leading to tumor inhibition in a poorly immunogenic TNBC mouse xenograft model and patient-derived tumor organoids. These results are promising for clinical application, but till now, there are no clinically approved exosome-based therapies. Further cohort studies are required to demonstrate the indicative role of exosomes in TNBC.

## Conclusion

sEVs are natural nano-sized extracellular vesicles with lipid membranes outside and bioactive contents inside. They generally can be secreted by almost all types of cells and play a critical role in intercellular signaling networks. They exhibit several properties such as targeted homing, stability, biocompatibility, low toxicity, and low immunogenicity. The distribution of various biological molecules including DNA, RNA, proteins, and cytokines within exosomes during physiological and pathological processes, including cancers, suggest that sEVs are involved in cancer occurrence and progression. sEVs derived from both tumoral and normal cells have emerged as important components of the tumor microenvironment. TNBC is a particularly aggressive subtype of breast cancer with earlier onset of metastatic disease, visceral metastases, rapid progression, short response duration to available treatment, and worse clinical outcomes. There is an urgent need to develop novel early diagnosis tools and therapies with good efficacy. sEVs have been shown to contribute to angiogenesis, immune escape, tumor proliferation, invasion and distant metastasis, and drug resistance in TNBC. In addition, sEVs can be easily isolated and detected in body fluids. Hence, they hold great promise as biomarkers for early diagnosis, prognosis, and treatment approach of TNBC.

The studies mentioned above provide the basis for the development of sEV-based biomarkers and therapeutics. It is also necessary to further explore the characteristics of sEVs, for instance, content sorting, transportation and internalization, circulation, and tissue clearance, to validate their role in the onset and development of TNBC. Moreover, answering the following questions may promote the clinical application of sEVs. Firstly, there have been no established standardized isolation and purification methods. Then methods such as miRNA quantification are not determined. Next, the precise mechanisms involved in the uploading of drugs into sEVs are unknown. Finally, the complexity of inclusions in sEVs may result in side effects and toxicity *in vivo*. There is still a need to conduct research and clinical studies on how sEVs participate in TNBC, as well as how to utilize sEVs in cancer diagnosis and treatment.

## Author contributions

The corresponding author is responsible for ensuring that the descriptions are accurate and agreed upon by all authors. The authors have contributed in multiple roles. YZ is responsible for writing the original drafts and literature search. ZX and WZ are responsible for literature search and editing for the original draft. All authors contributed to the article and approved the submitted version.

## Conflict of interest

The authors declare that the research was conducted in the absence of any commercial or financial relationships that could be construed as a potential conflict of interest.

## Publisher’s note

All claims expressed in this article are solely those of the authors and do not necessarily represent those of their affiliated organizations, or those of the publisher, the editors and the reviewers. Any product that may be evaluated in this article, or claim that may be made by its manufacturer, is not guaranteed or endorsed by the publisher.
